# An Energy-Friendly Scheduler for Edge Computing Systems

**DOI:** 10.3390/s21217151

**Published:** 2021-10-28

**Authors:** Alejandro Llorens-Carrodeguas, Stefanos G. Sagkriotis, Cristina Cervelló-Pastor, Dimitrios P. Pezaros

**Affiliations:** 1 Department of Network Engineering, Universitat Politècnica de Catalunya (UPC), 08860 Castelldefels, Spain; alejandro.llorens@entel.upc.edu; 2 School of Computing Science, University of Glasgow (UoG), Glasgow G12 8QQ, UK; s.sagkriotis.1@research.gla.ac.uk (S.G.S.); Dimitrios.Pezaros@glasgow.ac.uk (D.P.P.)

**Keywords:** fog computing, IoT, resilience, scheduling, single board computer, state of charge

## Abstract

The deployment of modern applications, like massive Internet of Things (IoT), poses a combination of challenges that service providers need to overcome: high availability of the offered services, low latency, and low energy consumption. To overcome these challenges, service providers have been placing computing infrastructure close to the end users, at the edge of the network. In this vein, single board computer (SBC) clusters have gained attention due to their low cost, low energy consumption, and easy programmability. A subset of IoT applications requires the deployment of battery-powered SBCs, or clusters thereof. More recently, the deployment of services on SBC clusters has been automated through the use of containers. The management of these containers is performed by orchestration platforms, like Kubernetes. However, orchestration platforms do not consider remaining energy levels for their placement decisions and therefore are not optimized for energy-constrained environments. In this study, we propose a scheduler that is optimised for energy-constrained SBC clusters and operates within Kubernetes. Through comparison with the available schedulers we achieved 23% fewer event rejections, 83% less deadline violations, and approximately a 59% reduction of the consumed energy throughout the cluster.

## 1. Introduction

Modern services, such as the Internet of Things (IoT), cooperative sensing, augmented reality, and Industry 4.0 have stringent requirements in terms of latency, availability, resilience and scalability [1]. To accommodate the latency requirements, the processing for these services is placed near the end users, at the edge of fog networks [2]. To satisfy the requirements for availability, resilience, and scalability, several nodes that inter-operate formulate clusters that enable failure-recovery and accumulative processing capacity.

Efficient management of the deployed clusters and seamless scalability is achieved by coupling network paradigms like edge computing (EC) and fog computing (FC) with containerization technologies and network function virtualization (NFV) [3]. Containers allow light virtualization by enclosing only the necessary code dependencies for the execution of a program. By using NFV, the various network functions (NFs), e.g., a firewall or a traffic control module, are deployed in a virtualized manner and can therefore be executed in a heterogeneous set of nodes. By using virtualization for the running NFs and the deployed services, the cluster of nodes becomes more versatile, and the deployment of services is simplified and therefore accelerated. Additionally, future migration to newer generations of hardware becomes significantly less time-consuming [4].

Single-board computers (SBCs) have become a mainstream choice for IoT environments [5]. In the past few years, the hardware capabilities of SBCs have improved significantly. Raspberry Pi 1 Model B launched in 2012 with a 700 MHz single core central processing unit (CPU). The current generation of Raspberry Pi has a 1.5 GHz quad-core CPU [6,7]. The available RAM has also grown from 512 MB to 8 GB. These changes have increased the adoption rate of SBCs as edge nodes for FC and EC, either as standalone devices or in clusters. This is evident by the variety of scenarios that utilize these devices: low-latency cyber-physical systems, resource-constrained computing, and next-generation data centers [8].

SBC clusters offer connectivity with a wide range of sensors and devices and therefore present new opportunities for the deployment of modern applications and services (e.g., connected vehicles, smart grids and e-health monitoring systems). Many use cases require location-specific measurements to meet latency-bound tasks, as the devices are co-located with a control device or within a monitored environment [9]. Such use cases were not easy to implement with previously available hardware, which would either provide good connectivity and reduced computational resources, or vice versa. Moreover, the small size of SBCs enables a higher density of devices, lower cost and higher energy efficiency when covering huge areas [6]. However, developing for such devices should always take into account the constraints in computational resources, which are not on par with more expensive conventional CPUs and full-scale computers.

Deploying and managing several applications in a cluster can be a laborious and error-prone task, even if the application is containerized. Therefore, a platform capable of orchestrating, managing, and ensuring the life cycle of containers and applications is necessary. One of the most prominent platforms for managing containers is Kubernetes [10]. Kubernetes encloses one or more containers within a different structure, called a pod, which is integrated in the rest of the Kubernetes API. Originally designed for data center environments, Kubernetes performs scheduling decisions based on key performance resources, e.g., CPU and memory utilization. Using these metrics, it enables safe rollbacks in case of failures and flexible scaling of the deployed pods. This, in turn, decreases the management complexity for the administrator of the cluster.

Kubernetes’ scheduling process, being primarily designed for data center environments, does not integrate energy measurements of the participating devices in the placement decisions. This can result in either under-utilizing the available energy resources or attempting deployments that are bound to fail due to insufficient resources. Moreover, the resources of the node that performs the scheduling decisions, known as the master node, are not included in the pool of resources for pod deployment. This results in under-utilized computational and energy capacity.

Given these shortcomings of a prominent orchestration platform, this paper proposes a scheduler that improves the resource utilization of energy-constrained SBC clusters by guaranteeing high levels of acceptance ratios, low overhead in the scheduling process, and better utilization of the available resources. To achieve these, we contribute the following:

- A regression model able to establish the relationship between battery consumption and used resources in an SBC cluster;
- A study of the impact of including the controller node in the scheduling process in an energy-constrained SBC cluster;
- A scheduling algorithm that assigns events to cluster nodes based on expected battery consumption and used resources;
- Real-world evaluation of the proposed scheduler on an energy-constrained SBC cluster.

The remainder of this paper is structured as follows. Section 2 describes the motivation of this work and related work. In Section 3, we present the problem statement and the notation and system model used in our approach. We introduce the proposed scheduling algorithm and explain the functions of its blocks in Section 4. Finally, we discuss the obtained results in Section 5.

## 2. Related Work and Motivation

This section provides a literature review of existing research works related to workflow scheduling in cloud computing. We have focused our attention on papers that consider energy as a primary factor in their solutions. In addition, our study analyzes several methods to estimate the state of charge (SOC) of battery-powered devices. This parameter is crucial for automating service deployment and extending the lifetime of the cluster.

### 2.1. Energy Efficient Scheduling

Although workload scheduling is a well-studied field in cloud and fog computing, there is a lack of practical implementation and prototypes in the reviewed literature. This work addresses this gap by testing the suggested solutions against a testbed comprised of representative SBC devices and batteries.

The authors of [11] have proposed a sleep schedule method based on compressive sensing to monitor environment data and device operating status. The proposed method arranges the working state of the nodes at different times to be in semi-sleep or sleep modes and selects the semi-sleep nodes at random in each cycle. Then, according to the gathered data by the active nodes, the data of the whole network can be reconstructed. However, the authors do not conduct any study on battery consumption to include this information in its scheduling decisions, which is a crucial parameter for WSN environments where most nodes are battery powered.

In [12,13], the authors propose energy efficient scheduling algorithms in a cloud computing environment to place tasks and reduce energy consumption. In both works, tasks are assigned to the appropriate VMs, and hosts are selected based on characteristics and current capacity. However, the authors of both papers use a simulation environment, i.e., the Cloudsim Toolkit [14], to evaluate their proposals instead of using a private cloud like Openstack [15] to demonstrate the effectiveness of their solutions in real scenarios.

A deep reinforcement learning (DRL) approach is used in [16] to address the task scheduling problem in fog-based IoT applications. The main function of its scheduler is to decide whether to process the task in a fog node or send it to the cloud data center. The authors include an energy consumption model in the scheduler’s proposal to guarantee selection of the most appropriate VM in terms of power consumption. Likewise, the authors of [17] have proposed a Q-learning algorithm to schedule tasks in an energy-efficient way. Their approach aims to minimize the task response time and maximize the utilization of a node’s CPU. By improving resource utilization, the authors reduce the energy consumption of the whole cloud system.

In [18], Varasteh et al. propose a framework to solve the power-aware and delay-constrained joint virtual network functions’ (VNFs) placement and routing (PD-VPR) problem. In the framework’s first phase, a centrality-based ranking method maps the VNFs to physical nodes. In a second stage, the delay budget between consecutive VNFs is split, and the shortest path through the selected nodes is found using the Lagrange relaxation-based aggregated cost (LARAC) algorithm.

Despite the plethora of papers addressing energy efficient scheduling algorithms in cloud and fog computing, they lack a practical evaluation using real-world testbeds comprised of resource-constrained nodes. All the approaches in the referenced works are implemented and evaluated in simulation environments, which require great CPU and memory capacity to execute and are therefore difficult to use and test.

To fill the identified gaps in literature, this paper proposes a scheduler which assigns events to resource-constrained nodes while guaranteeing low energy consumption and increasing resilience. The set of nodes that we used are off-the-shelf SBCs, typically found in use cases like unmanned aerial vehicles (UAV) and WSN. The proposed scheduler takes into account the controller node in the scheduling process, thus increasing the number of scheduled events and the acceptance ratio of the system. In this vein, our solution makes better use of the cluster’s available resources by considering not only the computing nodes but also the controller node.

### 2.2. State of Charge Estimation

In battery-powered devices, knowing the remaining battery capacity helps avoid service disruption due to battery depletion. To this end, several works aim to estimate battery *SOC* by using different methods.

In [19], Hu et al. present the extended Kalman filter (EKF) technique as a *SOC* estimation algorithm. The researchers evaluate the proposed estimator using two types of lithium-ion (Li-ion) batteries under different loading profiles and temperatures. The optimal model parameters used in the EKF are obtained from generic functions for battery modeling that combine several degrees of polynomials. Since the resulting model depends on the training datasets, the update of model parameters due to changes in these datasets may not be trivial in real-time scenarios.

The authors in [20] have proposed an enhanced coulomb counting method for estimating the *SOC* and the state of health (SOH) of lithium-ion batteries. They improve the estimation accuracy by considering the correction of the operating efficiency and the impact in the SOH. The authors’ proposed method can be easily implemented in all portable devices, such as SBCs, due to their simple calculation and low hardware requirements. Sagkriotis et al. [21] have also developed an application based on the coulomb counting method to monitor and reveal the energy profile of nodes that comprise an SBC cluster. Their outcome indicates the low-energy consumption characteristic of these devices while executing virtualized services. In [22], Pop et al. propose a new *SOC* algorithm by combining direct measurement of the electro-motive force (EMF) and Coulomb counting. They demonstrate the effectiveness of their approach by improving the *SOC* and accuracy of the remaining run time.

In contrast to the previous work, the authors of [23] have presented two methods for the actual bias modeling of batteries. They have proposed a polynomial and Gaussian process regression model using a typical battery circuit model to examine the bias modeling and the *SOC* estimation. The results of their model show a significant improvement in comparison with the baseline models (i.e., first- and second-order resistance-capacitance (RC) models) while being able to maintain similar computational efficiency.

The aforementioned papers have proven the accuracy of *SOC* estimation algorithms regarding battery capacity predictions. However, these papers use battery operation parameters such as current, voltage and temperature to make their estimations. The main focus in this paper is to establish a relationship between previous *SOC* measurements and utilization of resources (e.g., CPU) to train a regression model which predicts energy consumption. By doing so, we further contribute towards automating the placement process in such environments. By obtaining a model that is able to predict energy consumption, network-wide placement decisions are enabled. Such decisions can improve the total lifetime of the network and optimize the management of the available resources.

### 2.3. Single Board Computers

In the last years, the applicability of SBCs has increased and now covers a wide range of use cases. In [8], Johnston et al. perform an in-depth analysis of the state-of-the-art use cases for these devices. They explain the main characteristics of SBCs and detail the different device models. In addition, the authors identify the broad domains where the SBCs might be deployed. They emphasize how crucial these devices are as not only edge and fog nodes, because of their power requirements and size, but also as computational game changers that can bring computation closer to the data-generating parts of the network.

To improve the resilience and performance of SBCs, the authors in [9,21,24] have used SBC clusters. A cluster can be created either by coupling physical elements together or by using the concept of platform-as-a-service (PaaS) to create and manage the cluster. In [9], Bashford et al. present a new method for creating physical clusters of SBCs, called the Pi Stack. This method minimizes the amount of cabling required to create a cluster by reusing some elements of an SBC’s physical construction as a communication channel for both power and management. The researchers compare three different SBC clusters using the proposed technique. The clusters are composed of nodes from several vendors. Their results reinforce how important SBC clusters are as infrastructure for future IoT deployments. In contrast, Sagkriotis et al. [21] have explored the feasibility of a virtualized SBC cluster that can host scalable containerized applications. By using Kubernetes, they achieve resilience of the deployed energy monitoring application. They also demonstrate that an SBC cluster can host fog-oriented services. Likewise, Pahl et al. [24] have built an SBC cluster using the PaaS paradigm. However, they deploy their own dedicated tool to manage and configure the cluster rather than using a widely available platform, like Kubernetes. The authors do not compare against other management platforms. Thus, further evaluation must be done to demonstrate the feasibility and benefits of their approach.

Taking the PaaS concept and the above papers as a reference, we developed an SBC cluster to deploy services and tasks while adhering to their requirements. We extend the Kubernetes scheduler by including energy measurements and estimations to deploy services and tasks. We improved the cluster’s resilience by extending its lifetime while adapting Kubernetes energy-constrained devices.

## 3. Problem Statement and System Model

In this section, we formally present the problems we address as well as the notation and system model of use.

### 3.1. System Architecture

**Figure 1:**
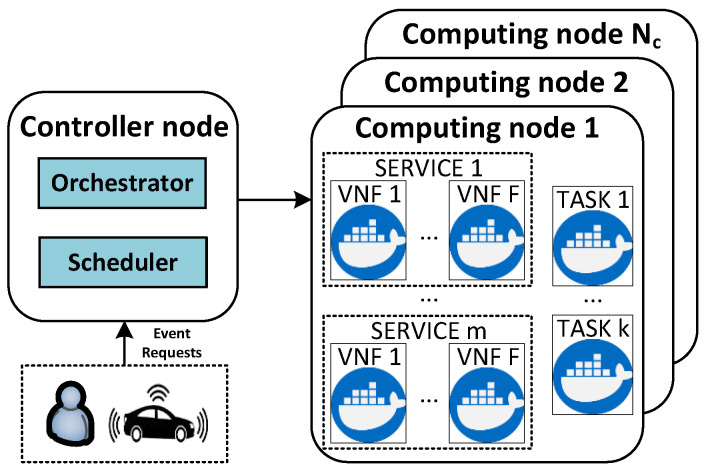
Reference architecture formed by a controller node and multiple computing nodes.

To capture the aforementioned environment characteristics and formally define the problems we investigate, we consider the reference architecture depicted in Figure 1. Under this architecture, a controller node is deployed alongside a set of computing nodes ($N_{c}$) in a commodity cluster of SBCs. Incoming event requests are scheduled and deployed according to their deadline and resource requirements. The computing nodes are considered to be energy-constrained devices, representative of edge nodes, and with a fixed amount of computational resources. Thus, the scheduler must place events by taking into account the remaining battery of each node with the ultimate goal of increasing the lifetime of the cluster and therefore its resilience. The controller not only must be capable of coordinating and managing the deployed services and tasks, but also of monitoring the battery status of each node, including its own battery levels.

### 3.2. Problem Modelling and Notation

We treat the cluster as a pool of available resources which has to be managed in a way that does not violate deadlines or energy constraints. We consider two types of events that are scheduled in the cluster: tasks and network services. The former is a set of instructions that require a pre-defined and fixed amount of time for their execution. Examples of tasks are: log rotation (the process of compressing log files that are older than a particular time and deleting ancient ones) associated with a particular running service, processing rows from a service database table, backing up a service database before its deletion, etc. The resources required for the execution of a task are released upon completion. A service event is composed of a set of VNFs that can reserve resources for an arbitrary amount of time that is not known in advance.

To include use cases that require fog-to-fog communication or other geo-distributed applications, the event requests are considered to be distributed among computing nodes that are geographically distant across multiple locations. An example use case would be on-boarding a set of drones to enable emergency service communications like telco services, such as IP telephony, between islands during a natural disaster (e.g., earthquake, fire, or flood). In this situation, each drone would run different VNFs: an access point (AP) VNF, a domain name system (DNS) VNF, and an access router VNF [25,26].

The incoming events are examined by the scheduler in an online manner, i.e., as they arrive and by examining the current resource capacity of the cluster. The events are scheduled to a set of deployable units within computing nodes (*P*). *P* represents all the virtual nodes (*p*) (the virtual nodes (*p*) are equivalent to Kubernetes pods) created on all the physical nodes where events can be scheduled (*N*). Each virtual node p∈P is identified with an ID. The parameter $p_{i}^{n}$ indicates that virtual node $p_{i}$ is placed in physical node *n*, where n∈N. Any given network service (*S*) is formed by a sequence of VNFs (*F*), where each function *f* must be processed on a set of physical nodes. These functions must be scheduled one after the other in a specified sequence. Each created virtual node *p* can only process one function at a time. Similar to the network functions, any given task (*T*) will be processed on the selected physical node where a virtual node *p* will be created to execute the requested task. Each event has a demanded rate ($r_{e}$) that must be met by the selected node. In addition, the processing capacity of the node ($c_{n}$) has to cover the demanded rate by the event (i.e., $c_{n}\geq r_{e}$).

All the network functions and tasks have a running time parameter ($t_{r}$) that denotes the amount of time that must pass before an event can be considered completed. When $t_{r}>0$, the event runs during the specified time. In the case that this parameter is 0, or not specified, the event will be executed during the whole life cycle of the system. Both types of events also have a starting time ($t_{s}$) and a completion time ($t_{c}$). The former represents the precise moment when the selected node starts processing the event. The latter specifies when the network functions or the tasks finish execution of the events. Thus, we can calculate the execution time ($t_{e}$) of an event through the following equation:

(1) $$t_{e}=t_{c}-t_{s}$$

In addition, the event’s time of arrival ($t_{a}$) is registered to calculate the whole time that an event is in the system ($t_{t}$). Thus, the first parameter ($t_{a}$) denotes the time when the request for scheduling was received by the controller node. The second parameter ($t_{t}$) is defined as the total time starting from when an event arises until its completion, and it can be calculated as follows:

(2) $$t_{t}=t_{c}-t_{a}$$

As our system will receive event requests with an unknown arrival time, we introduce a priority queue to the controller node. The events can have different priorities according to user demands. The priority queue works as a centralized event allocation system to coordinate several nodes. While the events are ordered in the queue according to certain criteria, the scheduler takes the highest element in the list and chooses the best physical node to deploy a virtual node that will run the event (task or network function). The priority queue introduces a certain delay in the assignation node procedure, since the controller node schedules events one by one. In the case of the arrival event rate becoming higher than the scheduling rate, we define the waiting time ($t_{w}$) of an event as the amount of time from its arrival until its execution is started. This parameter can be obtained from the following equation:

(3) $$\begin{array}{c} t_{w}=\left\{\begin{array}{cc} t_{s}-t_{a_{f=1}} & \;\mathrm{if}\;\mathrm{the}\;\mathrm{event}\;\mathrm{is}\;a\;\mathrm{service}\\ t_{s}-t_{a_{T}} & \;\mathrm{if}\;\mathrm{the}\;\mathrm{event}\;\mathrm{is}\;a\;\mathrm{task}\; \end{array}\right. \end{array}$$

From Equation (3), we obtain the waiting time for each event. In the case of a service, we need the arrival time of the first network function, as this function represents the beginning of the service. Both the waiting and total time parameters are considered evaluation metrics that capture the performance of the scheduler.

Finally, a deadline (*d*) is defined for processing a given event. In the case of a network service, the processing of its last function must be completed by this time. Otherwise, the scheduler incurs a service level agreement (SLA) violation. A list of notations related to the system model is provided in Table 1.

## 4. Proposed Scheduling Solution

**Figure 2:**
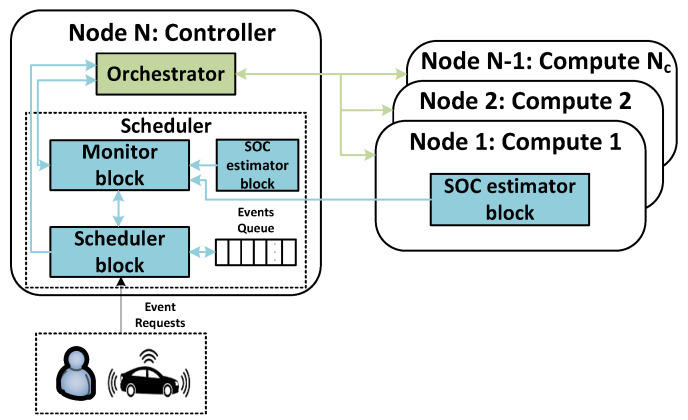
Scheduling module design and the relation among its different blocks.

In this section, we propose our *SOC* and capacity-based scheduler (SOCCS). It processes event requests and determines the best node in an SBC cluster to run them based on the remaining battery estimations and CPU usage in the nodes. The proposed scheduler is formed by three main elements: the *SOC* estimator, the monitor and the scheduler. The relation among these blocks and how they communicate with the orchestrator (represented by blue lines) as well as the communication between the orchestrator and the cluster elements (represented by green lines) is depicted in Figure 2. Please note that in this representation, the set of nodes where events can be scheduled (*N*) comprises the controller node and the computing nodes ($N=N_{c}+1,N_{c}\subset N$). The functions of each block in the scheduling module are explained in the following subsections.

### 4.1. *SOC* Estimator Block

This element acts as an agent since it runs in every node comprising the SBC cluster. Its main functions are to receive the necessary information from the measurement equipment and calculate the *SOC* using the coulomb counting method [20].

The *SOC* estimation can be dependent on certain characteristics of the batteries (e.g., state of health and model) by taking into account the used estimation model [27]. Such factors are related to the battery hardware. Thus, the estimators have to be updated when there is a change in the hardware. The battery hardware-dependent estimations are less evident in the coulomb counting method, as it monitors the total electric charge that a battery absorbs or releases during its charging or discharging phases.

The estimation of the *SOC* can be achieved by dividing the percentage of the released electric charge in the battery by the one that entered it. Denoting the released capacity when the battery is completely discharged as $Q_{releasable}$ and the rated capacity as $Q_{rated}$, the *SOC* percentage can be obtained as follows:

(4) $$SOC=\frac{Q_{releasable}}{Q_{rated}}\cdot 100\%$$

The proposed estimator block adopts the coulomb counting method because of its simple yet accurate approach. To get more accurate results, the $Q_{rated}$ term is obtained by considering the actual electric charge that the battery can deliver over several charge-discharge cycles. Following a similar methodology as the one proposed in [20], we can find the coulombic efficiency (η) of the rated capacity. Additionally, we used the maximum releasable capacity ($Q_{max}$). Thus, Equation (4) is adjusted to:

(5) $$SOC=\frac{Q_{max}}{\eta \cdot Q_{rated}}\cdot 100\%$$

When the battery is fully charged, the *SOC* is given by Equation (5). However, during a discharging phase, we should know the percentage of the capacity relative to the $Q_{rated}$ term, denoted as the depth of discharge (*DOD*). The DOD is obtained from a measured charging and discharging current ($I_{b}$) in an operating period τ and then subtracted from the total SOC as shown the following equations:

(6) $$\begin{array}{c} \Delta DOD=\frac{-\int_{t_{0}}^{t_{0}+\tau }I_{b}\left(t\right)\;dt}{\eta \cdot Q_{rated}}\cdot 100\% \end{array}$$

(7) $$\begin{array}{c} DOD\left(t\right)=DOD\left(t_{0}\right)+\Delta DOD \end{array}$$

(8) $$\begin{array}{c} SOC(t)=100\%-DOD(t) \end{array}$$

The DOD is an accumulated value as shown in Equation (7). We can estimate the *SOC* of the battery through Equation (8) at any time. The estimation process is based on the measured voltage and current. The *SOC* estimator block knows the battery operation mode from the value and direction of the operating current. During the discharging phase, the DOD adds up the drained charge until reaching the $Q_{max}$ value when the battery is exhausted (i.e., SOC = 0%). Meanwhile, the DOD counts down the accumulated charge in the charging phase until the battery is fully charged (i.e., SOC = 100%).

The *SOC* estimator block uses the aforementioned procedure to estimate the *SOC* battery in each node where it is running. Finally, it sends the estimated value to the monitor block.

### 4.2. Monitor Block

This module is responsible for monitoring all the virtual nodes that have been created and assigned an event request by the scheduling algorithm described in Section 4.3. It also monitors the status and usage of the physical nodes by communicating with the orchestrator. Specifically, the monitor module tracks the CPU and memory consumption of each node, as summarised in Procedure 1.

**Procedure 1:** Update Nodes.

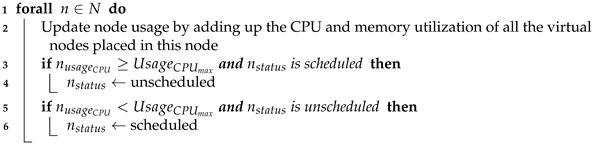



This procedure updates the resource utilization of each node within the SBC cluster. It determines a node’s resource utilization by calculating the whole usage of its virtual nodes in terms of CPU and memory (line 2). Considering all the cluster nodes as candidates to place a created virtual node by default, this procedure checks if a current node’s utilization has not reached its defined maximum capacity (line 3). If the maximum capacity has been reached, the node’s status is marked as *unscheduled*, and it is excluded from the candidate selection process in the scheduling algorithm (line 4). Line 5 checks for the opposite condition. It verifies that the current node’s usage is below its maximum value. The node’s status is set to *scheduled* in line 6 if it was previously marked as *unscheduled*. The updating nodes procedure is used by the monitor block, and its behavior is described in Algorithm 1.

The monitor block’s procedure begins by initializing two parameters (lines 1–2) that run during the whole lifetime of the system (line 3). It checks several parameters (e.g., $p_{status}$, $p_{t_{c}}$, $p_{d}$) for all the created virtual nodes (p∈P) to verify if certain conditions have been satisfied. If a virtual node is running, the monitor block gathers its CPU and memory usage from a metrics server (e.g., Prometheus) and records these values (lines 5–7). Otherwise, the event is determined to be in one of two possible states: *succeeded* or *failed*. In the case that an event has completed its execution, the virtual node where it was running is marked as succeeded. If the event has been completed past its deadline (line 9), the algorithm updates the amount of deadline violations in line 10. The other state is related to the failed virtual nodes (line 11). When this condition is satisfied, the virtual nodes monitor updates the amount of rejected events in line 12. Afterwards, the algorithm releases the used resources and updates the respective parameters (lines 13–15). Finally, the algorithm calls the updating nodes procedure in line 16. The rejected events and deadline violations parameters are later used as evaluation metrics of the proposed scheduler. The metrics are analyzed in detail in Section 5.

Finally, the monitoring block receives the *SOC* battery information sent by the *SOC* estimator block in a parallel process to Algorithm 1. The received *SOC* information is saved in $n_{usage_{SOC}}$. As a result, the scheduling block is able to obtain the utilization of a node in terms of CPU usage, memory usage and *SOC* by reading the stored values in $n_{usage}$.

**Algorithm 1:** Monitor Process.

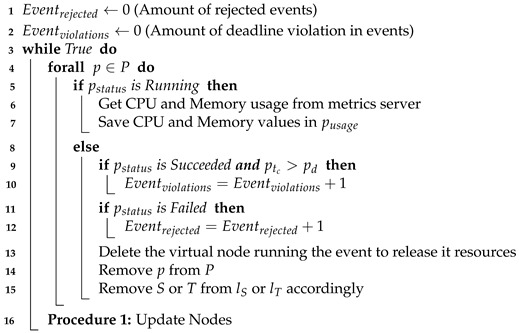



### 4.3. Scheduler Block

This module determines the best node where an event can run according to the *SOC* prediction. The scheduler block receives the event requests one after the other and appends them to a priority queue. At the same time, it takes the events from the priority queue one by one and determines the node where each event will run based on the remaining battery estimations and CPU usage. The *SOC* prediction is determined through a regression model [28] that is explained in Section 4.3.1, and Procedure 2 summarises the *SOC* prediction methodology used by Algorithm 2.

**Procedure 2:***SOC* prediction.

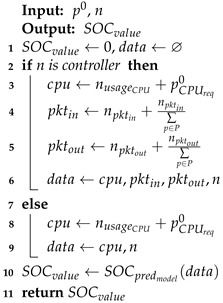



Procedure 2 uses a trained prediction model to forecast the *SOC* value that a node would have if a specific virtual node that is running an event were assigned to it. The procedure takes as input objects the current node and the virtual node to be scheduled. Then, it extracts and determines the required information by the *SOC* regression model to predict the *SOC* value. The first step in the *SOC* prediction procedure is to initialize the output variable and create an empty set to store the data used by the prediction model (line 1). Next, the procedure checks if the current node is the controller node, because the data used by the model is determined by the node’s type (line 2). In Section 4.3.1, we explain why we make this differentiation in the input data to the model. Lines 3–6 determine and store in the data variable the values required by the model in the case of the controller node. In line 3, the expected CPU usage of the node, if the virtual node were deployed to it, is calculated by adding the current CPU usage and the required CPU of the event running in the virtual node. Lines 4 and 5 determine the expected overall number of exchanged packets between the controller and the computing nodes once the virtual node is scheduled. The obtained values are then stored in the data set (line 6). In the case of computing nodes, the procedure only factors in the expected CPU usage and saves this value in the data set (lines 7–9). Finally, the prediction value is obtained from the *SOC* regression model considering the stored data (line 10).

Algorithm 2 is executed during the whole lifetime of the scheduling algorithm while there are existing elements in the priority queue (line 1). Before analyzing any possible node to assign an event, the algorithm obtains the node’s capacity from the monitor block (line 2). After gathering the usage of the nodes, the algorithm takes the first element in $l_{priority}$ and initializes the list of possible candidates to host the new virtual node (lines 3–5). The purpose of the following steps in this algorithm is to define the potential candidate SBCs where virtual nodes can be placed. Each physical node with a battery percentage above a minimum predefined value and a scheduled status is added to $l_{candidate}$ (lines 6–8). After that, the algorithm removes the controller node from the $l_{candidate}$ list if it is present in the list and there is at least one computing node available (lines 9–10). Thus, the algorithm increases the controller’s longevity and avoids associated extra processing for deploying an event on it.

If the previous condition is not met (line 9), we analyze two possibilities with the same outcome (line 11). The first possibility corresponds to the case when no node can host a virtual node. The second possibility is more complex, since the only available node is the controller and the event to schedule will run during the whole lifetime of the system. For both cases the associated event (i.e., service or task) is rejected in lines 12–22, and $Event_{rejected}$ metric is updated. Accordingly, the created virtual node for that event is removed to release its resources, and *P*, $l_{S}$ and $l_{T}$ are updated. In the case of a service event, the algorithm checks each former network function. If the function has already been deployed, it is deleted to release the associated resources. In the case that the function is in the priority queue, it is also removed so as to not be considered by the scheduler and save further resources. After not meeting the aforementioned conditions (lines 9 and 11), we have at least one node in $l_{candidate}$ (line 23). Notice that the controller node can be in $l_{candidate}$ when there are no more available computing nodes and the events to be placed have a specified running time ($t_{r}>0$). In this way, our proposal scheduler reduces the number of rejected events, as demonstrated in Section 5.

The process of Algorithm 2 continues, and in the next step the first element in $l_{candidate}$ is assumed as the best node (line 24). This node is then analyzed to determine if any candidate node has a better score than this current best node (lines 25–32). In line 26, the algorithm verifies if the *SOC* predictor model exists. If it does, the predictor model calculates the *SOC* of the current candidate node using Procedure 2 (line 27).

The output of the *SOC* prediction procedure is used in Algorithm 2 to calculate the node score through Equation (9) (line 28). With this equation, the algorithm tries to maximize the node score by selecting the node with the highest *SOC* and the minimum CPU usage.

(9) $$n_{score}=\alpha_{1}\cdot \left(SOC/100\right)+\left(1-\alpha_{1}\right)\cdot \left(1-E_{\mathrm{CPU}}/Usage_{{\mathrm{CPU}}_{max}}\right)$$

In Equation (9), the value $\alpha_{1}$ is an adjustable positive weight with values between 0 and 1. The *SOC* term can correspond to either a predicted value (i.e., using the *SOC* regression model) or a real measured value from the *SOC* estimator block. The expected CPU usage ($E_{\mathrm{CPU}}$) represents the new CPU usage that the analyzed node would have if a virtual node running an event were scheduled to it. This value is calculated by adding the current CPU usage of the node and the required CPU usage of the event. The steps are similar to the one previously described, but the node score is calculated using the current *SOC* of the node when the *SOC* predictor model is not available (lines 29–30). After calculating $n_{score}$, Algorithm 2 checks if this value is higher than the best node score (line 31). In the case that the value is higher, the current node is taken as the new best node (line 32). Finally, the scheduler block communicates to the orchestrator to bind $p^{0}$ in $n^{best}$ (line 33).

**Algorithm 2:** Event Scheduler.

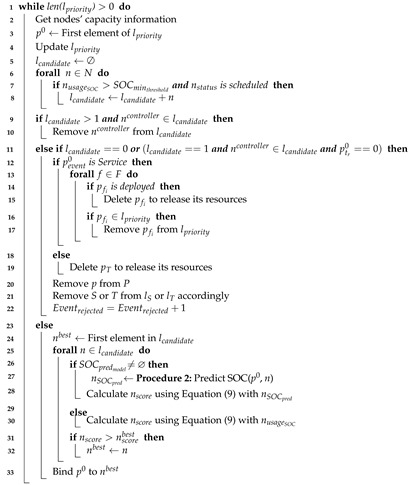



Algorithm 3 represents the main process of the scheduler application which initializes the other processes, i.e., the monitor process, the event scheduler and the regression model handler (see Section 4.3.1) (lines 5–7). The algorithm begins by initializing the set of virtual nodes, which can be updated by both the scheduler and monitor block, and the event lists (lines 1–3). Additionally, it initializes the *SOC* prediction model used in Algorithm 2 and the data set to train the model in Algorithm 4 (line 4). The data_training variable is updated in each cycle of the Algorithm 1. Algorithm 3 waits for any event request while the scheduling application is running (line 8). When a request arrives, the algorithm adds it to the corresponding list in line 9. Then, it creates a virtual node with the requirements for the event and adds it to the set of virtual nodes (line 10). After creation of the virtual node, the events’ ranking in the priority queue ($l_{priority}$) (lines 11–13) is determined by a process that considers two factors: delay(p) and $wait_{queue}\left(p\right)$. The former represents the amount of time that the scheduler can delay the execution of an event without missing its deadline (see Equation (10)). The latter is the waiting time of the event before being processed by the scheduler (see Equation (11)). In both equations, we denoted the current system time as $t_{now}$. Note that the smaller the delay(p), the faster the created virtual node will be executed.

(10) $$\begin{array}{c} delay\left(p\right)=p_{d}-t_{now}-p_{t_{r}} \end{array}$$

(11) $$\begin{array}{c} wait_{queue}\left(p\right)=t_{now}-p_{t_{a}} \end{array}$$

Based on the previous definitions, we calculate the ranking score for the virtual node where an event runs, denoted by $p_{rank}$, as follows:

(12) $$p_{rank}=\beta_{1}\cdot delay\left(p\right)-\left(1-\beta_{1}\right)\cdot wait_{queue}\left(p\right),$$

where $\beta_{1}$ is an adjustable positive weight with values between 0 and 1. A virtual node with the lowest ranking must be executed first. Thus, the algorithm updates the priority list and sorts the queue by taking into account the calculated ranking of the virtual node (lines 14–15).

**Algorithm 3:** Main process.

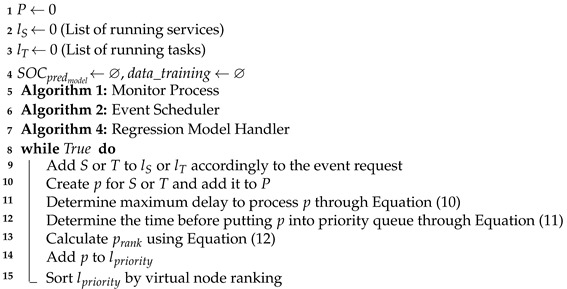



#### 4.3.1. *SOC* Regression Model

In contrast to the described methodology in Section 4.1, which is under the umbrella of direct calculation methods and model-based methods, data-driven methods do not require an equivalent circuit or electrochemical mechanism model to describe battery behaviors. Thus, the data-driven methods can estimate the battery *SOC* through sampled data by finding a relation between the data and the *SOC* measurements. Methods of this kind include autoregression moving average (ARMA), artificial neural network (ANN), support vector machine (SVR) and others [29]. These methods can cause a large computational burden when the training data is huge. Additionally, they must be trained in an initial state before their hyper-parameters can be adjusted. Thus, these methods might not be feasible for use cases where an SBC battery-powered cluster must also process service and task requests.

In the case of regression models, the model coefficients are determined from available training data by minimizing the root mean square error (RMSE) between the predicted and real values. The RMSE represents the standard deviation of the prediction errors, thus showing how concentrated the data is around the line of best fit [28]. In general, regression models can be classified into two types: polynomial and linear regression models. The former may include higher powers of one or more predictor variables and is defined in Equation (13). The latter may include the interaction effects of two or more variables and represents an example of a multiple linear regression model. It is represented in Equation (14) [28].

(13) $$\begin{array}{c} y=\beta_{0}+\beta_{1}x+\beta_{2}x^{2}+\ldots +\beta_{k}x^{k} \end{array}$$

(14) $$\begin{array}{c} y=\beta_{0}+\beta_{1}x_{1}+\beta_{2}x_{2}+\beta_{12}x_{1}x_{2}+\ldots +\beta_{mn}x_{m}x_{n} \end{array}$$

In this paper, we adopt a regression model for *SOC* estimation, since the computational characteristics of SBCs might not be capable of supporting complex algorithms, such as ARMA, ANN and SVR. The regression model is trained with an initial dataset formed by collected metrics during a defined period. To improve its accuracy, it is updated when the RMSE metric is above a pre-defined threshold. The model coefficients are calculated and updated through Algorithm 4.

**Algorithm 4:** Regression Model Handler.

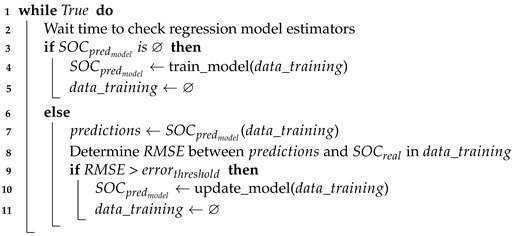



This algorithm checks the *SOC* regression model after waiting a pre-defined time (line 2). After that, it verifies if the regression model has not been training (line 3). Then, the model is trained with the existing training dataset (data_training), and this dataset is initialized to gather new data for future model examinations (lines 4–5). If a model already exists (line 6), the algorithm studies the model’s coefficients to determine if they must be updated (lines 7–11). Using the existing gathered data, the algorithm makes several *SOC* predictions from the trained model (line 7). Then, it determines the RMSE between the predictions and the measured *SOC* in data_training (line 8). In line 9, the calculated RMSE is checked to determine if it is above a pre-defined threshold. If this is the case, Algorithm 4 updates the *SOC* regression model using the existing dataset (line 10). After this process, the training dataset is restarted, and newly gathered data is added through the monitoring process (line 11).

## 5. Evaluation and Results

**Figure 3:**
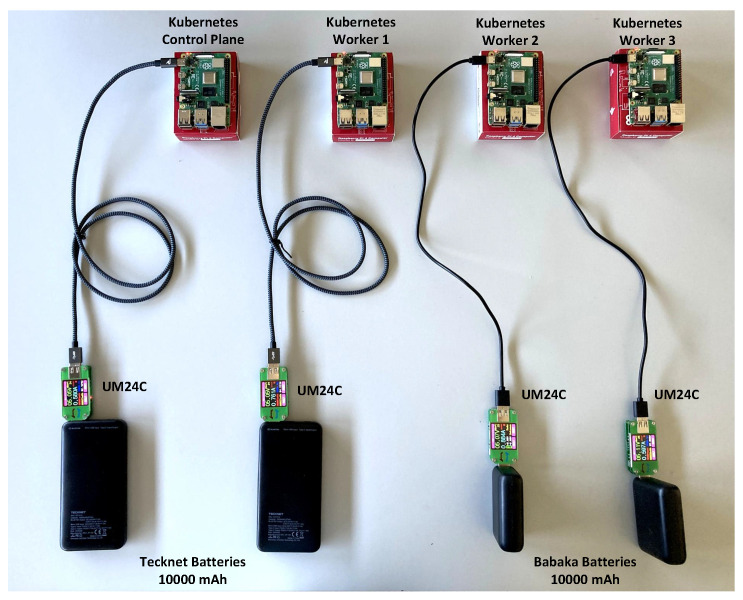
SBC cluster using Raspberry Pi devices with Kubernetes running on top of them.

To evaluate the performance of our proposed scheduling algorithm, we have used a testbed formed by a cluster of four Raspberry Pi 4 Model B units [7]. These devices represent regular IoT devices that can connect with a variety sensors and offer edge processing capabilities. The sensor device used to measure the power consumption of each node was the UM24C module [30], which connects to the Raspberry Pi devices via Bluetooth. The energy sources for the Pi devices are batteries with a capacity of 10,000 mAh. Figure 3 depicts the described testbed. We have deployed Kubernetes 20.04 as the management framework for virtualized services and tasks. Services and tasks run as docker containers within pods. The deployed events are placed into the devices and utilize their available capacity according to predefined requirements. The proposed scheduling algorithm was implemented using Python 3.6.8 and deployed within Kubernetes, replacing the baseline scheduler.

In our evaluation scenarios, the services and tasks to be scheduled arrive one at a time following a Poisson distribution. We explore different event arrival rates that range from 2 to 12 events per time unit. The main parameters used for creating the services and tasks are selected randomly from the list of values shown in Table 2 following a uniform distribution. The evaluation parameters are defined based on typical workloads derived from the literature. Notably, the CPU usage is measured in CPU units and is expressed as an absolute quantity. Thus, 100 milliCPU and 0.1 CPU are the same amount of CPU usage in a single-core, dual-core, or 48-core machine.

### 5.1. Utilizing Unused Controller Resources

This section analyzes the impact of deploying selected events to the controller. Different from previous works in cloud and edge computing ([13,31,32]), this work includes the controller node in the scheduling process to deploy service and task requests. The reviewed papers do not consider deploying events in the controller node because they speculate that this will cause additional processing overhead in the controller and an increase of the total time of the events in the system. However, by selecting events that have a fixed execution time, we can reduce the negative effects of deploying them in the controller. More specifically, the controller is able to host a new event using the released resources of the previous event, therefore increasing the amount of overall scheduled events and the overall acceptance ratio. Thus, our scheduler effectively uses all the available resources in the cluster.

**Figure 4:**
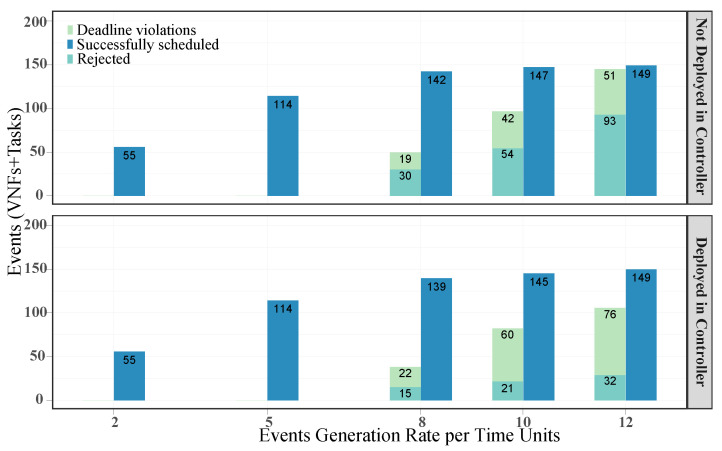
Number of deadline violations, rejected events and successfully scheduled events.

To study the impact of this decision, we ran several experiments for both types of scheduling (including/excluding the controller). The results are presented with a confidence interval of 95%. Figure 4 depicts the average number of successfully scheduled and rejected events as well as deadline violations for different event generation rates. We considered successfully scheduled events those that did not exceed their deadlines. We assume that surpassing a deadline would not lead to a task or service interruption, since maintaining a required QoS is a desirable parameter but not mandatory [16].

Notably, the number of successfully scheduled events was relatively similar for both criteria, i.e., deploying and not deploying in the controller node, and all the event generation rates. However, there is a noticeable difference in the number of rejections and deadline violations for generation rates of 8, 10 and 12. For the case of services and tasks that are assigned to the controller, we observe a reduction of 50%, 61% and 66% in the amount of rejected events, respectively. These reductions are more significant than the increments in the deadline violations, which can be up to 16%, 42% and 49% for the same generation rates.

**Figure 5:**
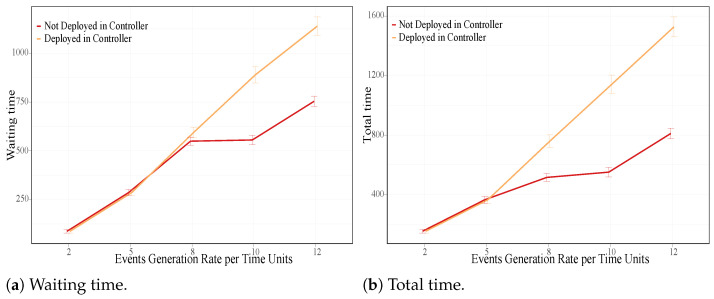
Average metrics time while deploying or not deploying events in the controller node.

Figure 5a shows the average waiting time of the events for both criteria. We notice that the waiting time increased when the events were deployed to the controller (soft orange line) with regards to the other criterion (strong red line), since they were placed one by one using the resources released by the previous events. Consequently, no extra load was added to the controller, and possible saturation in the system was thus avoided. Similar results were obtained for the average total time of the events, which includes the waiting time, as seen in Figure 5b.

**Figure 6:**
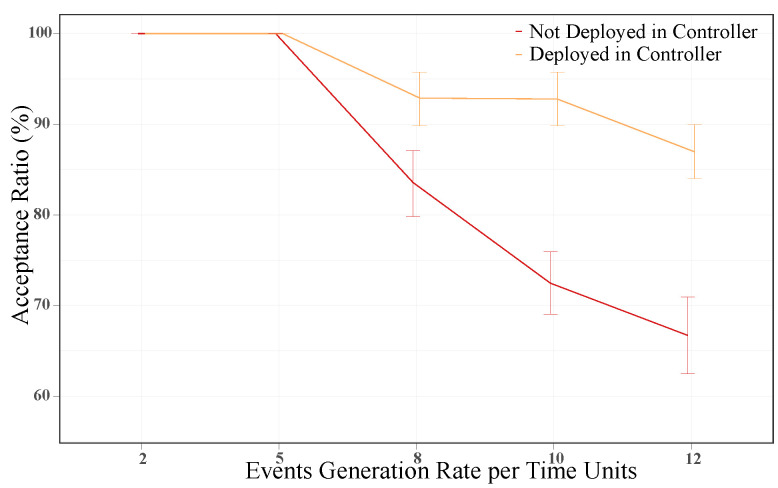
Average event acceptance ratio with and without deploying events in the controller node.

By using the controller node to deploy selected services and tasks, we minimize rejections and effectively utilize all available resources. In Figure 6, we show the average acceptance ratio of events for both criteria. For a generation rate of 2 and 5 events per time unit, both criteria have an acceptance rate of 100%. However, for generation rates greater than 5 events per time unit, the performances were different. Specifically, when deploying events in the controller node, the event acceptance ratio was increased by 11%, 28% and 30% for generation rates of 8, 10 and 12 events per time unit, respectively.

Based upon these results, we can confirm that using the controller node to deploy specific events guarantees a higher event acceptance ratio than using its resources solely for scheduling tasks. The higher acceptance ratio was evidenced by a significant reduction in the number of rejected events, although it was at the cost of greater deadline violations. Overall, the advantages outweigh the drawbacks when deploying services and tasks in the controller node in a resource-constrained environment. Thus, the proposed scheduler has been implemented by taking these results into account.

### 5.2. *SOC* Regression Model

**Figure 7:**
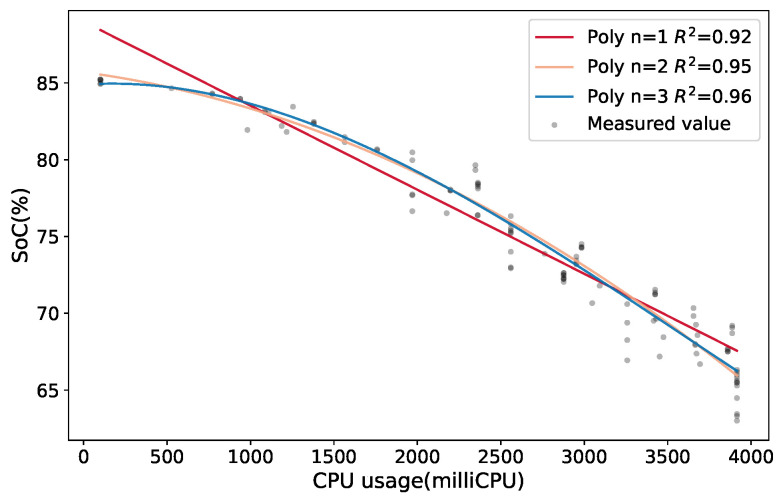
Multiple regression models for the *SOC* estimation based on CPU usage for a computing node.

Before starting the training phase, several regression models were studied to choose the one that best fit our case of study. Figure 7 shows three polynomial models of first, second and third orders which use the CPU usage as a predictor to estimate the *SOC* in a computing node. To describe the accuracy of the models, we included in the graphic the adjusted $R^{2}$ parameter which reflects the variation in the number of predictors. Additionally, the adjusted $R^{2}$ parameter does not automatically increase when more predictors are added to the model. According to this metric, the best model was the third-order model, which is represented by the blue line. Moreover, given the negligible accuracy difference of the third-order model and the computation overload it requires, we used the second-order polynomial to strike a balance between accuracy and computation cost.

**Figure 8:**
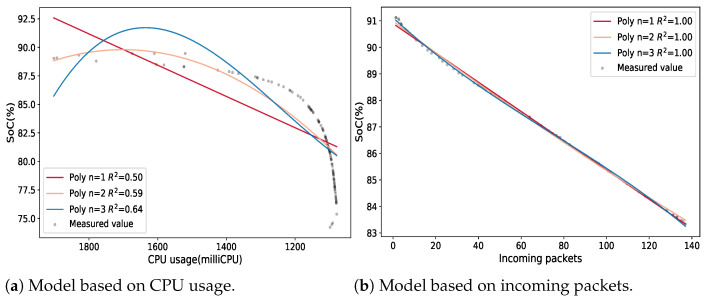
Multiple regression models of the *SOC* for the control plane node.

The controller node has a different behavior with regards to the computing nodes, since its main function is to schedule service and task requests. This does not demand as much CPU usage as the deployment of services and tasks. Figure 8a depicts the controller’s behavior when the CPU usage reaches its higher value of around 1900 milliCPU and starts to decrease while the *SOC* begins to decline. From the adjusted $R^{2}$ values, we can see that none of the analyzed models, based on CPU usage, fit the data correctly because the values were below 0.80. In response to this result, we analyzed another metric different from CPU usage. The selected metric was the number of incoming packets in the controller node since it receives user requests and worker messages. The studied polynomial models are shown in Figure 8b. This figure shows that all the models perfectly fit the data, as their adjusted $R^{2}$ was 1.

**Figure 9:**
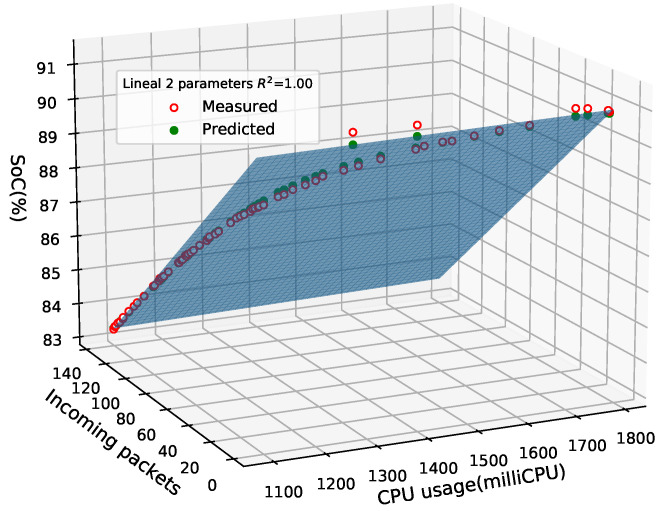
Linear regression model with CPU usage and incoming packets as predictors for the controller node.

The CPU usage represents a boundary parameter for the SBCs, because when a node reaches its maximum value, it cannot process new requests which would affect its performance. As a result, we must also consider CPU usage in the regression model for the controller node. In this regard, Figure 9 depicts a three-dimensional representation of the regression model for the controller node based on incoming packets and CPU usage. When we have two predictors, the least square regression line becomes a plane with two estimated slope coefficients. The model’s coefficients are estimated by finding the minimum sum of squared deviations between the blue plane and the measured values. With consideration of the adjusted $R^{2}$, this model fits our data, since it has the highest possible value.

Finally, the *SOC* regression model for any node *n* in the SBC cluster (n∈N, where $N=N_{c}+1$) can be expressed as follows:

(15) $$SOC_{pred_{model}}\;=\delta_{0}+\delta_{1}\;\cdot \;pkt_{in_{n}}\;\cdot \;ctrl+\delta_{2}\;\cdot \;cpu_{n}+\;\;\sum_{\forall i\in N_{c}}\left(\delta_{3_{i}}\;\cdot \;compute_{i}\right)\;\cdot \;cpu_{i}^{2}+\delta_{4_{i}}\;\cdot \;compute_{i}$$

The model coefficients (δ) were obtained from the available training dataset. We introduced the categorical variables $compute_{i}$ to represent each computing node in the model ($i\in N_{c}$). These variables are binary. The node whose *SOC* is to be predicted takes a value of 1 and the others take a value of 0 (e.g., $compute_{1}$ = 1, $compute_{2}$ = 0, …, $compute_{N_{c}}$ = 0). Notice that in the case of the controller node (n=N), all the categorical variables are 0 (e.g., $compute_{1}$ = 0, $compute_{2}$ = 0, …, $compute_{N_{c}}$ = 0). Regarding the $pkt_{in}$ term, we added a binary indicator ctrl to distinguish if the node is a controller or not. Thus, it takes a value of 1 when the controller’s *SOC* will be predicted, otherwise it will be 0. Thus, our *SOC* regression model is transformed into a linear model with two predictors (i.e., $pkt_{in_{n}}$ and $cpu_{n}$). In contrast, for the computing nodes, a second-order polynomial model based on CPU usage was used, since the $pkt_{in}$ term was discarded due to its low value with regards to the CPU usage.

### 5.3. Comparison among Scheduling Algorithms

To the best of our knowledge, no other online scheduler is built for improving the acceptance ratio of requested events in an energy-constrained SBC cluster. Therefore, we compared our proposed scheduling algorithm, SOCCS, with the well-known greedy least loaded scheduler (LLS) and the native Kubernetes scheduler (KS). The former algorithm aims to allocate the different events to the node with the highest available buffer capacity. Thus, the node with the least CPU usage is chosen. The latter algorithm ranks the feasible nodes and selects the one with the highest-ranking score. In other words, the more resources the services and tasks use in a node, the lower its ranking is. These algorithms were analyzed through the following metrics: scheduled and rejected events, deadline violations, waiting and total time, acceptance ratio and battery consumption. To ensure the reliability of the results, we ran several experiments for each generation rate for all the analyzed scheduling algorithms and show results with a confidence interval of 95%.

#### 5.3.1. Average Number of Scheduled and Rejected Events

**Figure 10:**
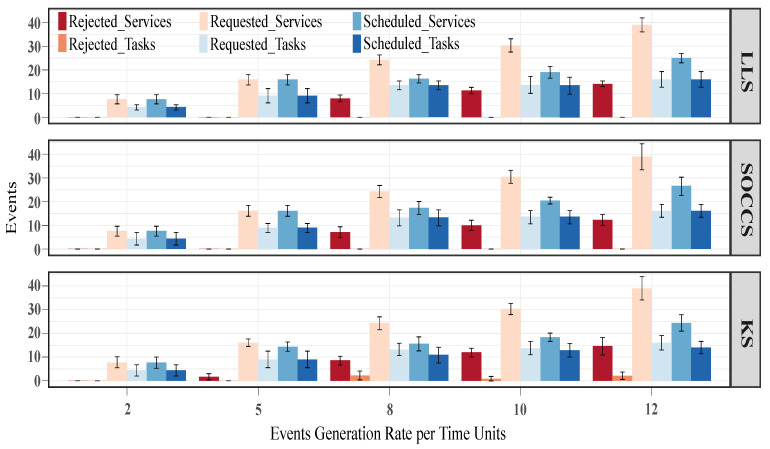
Number of requested, scheduled and rejected events (i.e., services and tasks) for all the scheduling algorithms.

Figure 10 depicts the obtained results in terms of requested, scheduled and rejected events, i.e., tasks and services. We can observe that all the schedulers, except for the KS, achieved similar results when the generation rate was small (i.e., 2 and 5), since they deployed all the requested services (rejected services were 0). The KS had around 2 rejected services (red bar) for a generation rate of 5 events per unit time. We also note that for high generation rates (i.e., 8, 10 and 12), KS rejected around 2 tasks (salmon bar). In contrast, the other algorithms deployed all the requested tasks (mid blue bar).

**Figure 11:**
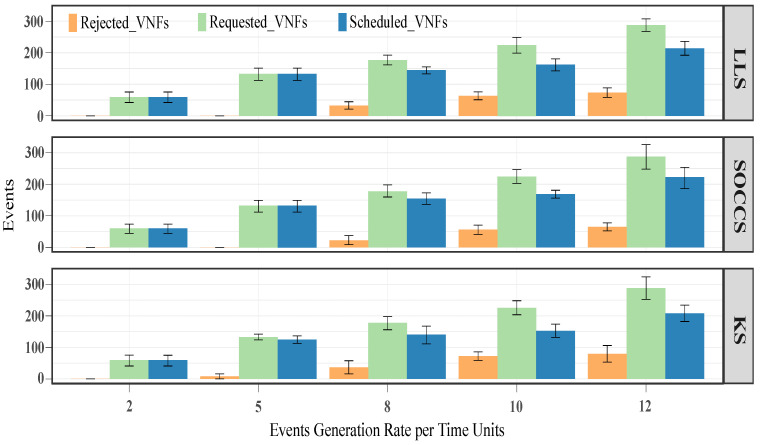
Number of requested, scheduled and rejected VNFs for all the scheduling algorithms.

Since the generated services are formed by several network functions (VNFs), Figure 11 shows the obtained results for their constituent VNFs. By analyzing the rejected VNFs for low-value generation rates, we appreciate that KS rejected around 8 VNFs (apricot bar) for 5 events per unit time. By contrast, the other schedulers were able to schedule all the requested VNFs when the generation rates had low values. In terms of scheduled VNFs (windows blue bar), our scheduler outperformed the LLS and KS algorithms by 19% and 17%, 8% and 7%, and 5% and 2%, respectively, for the high-value generation rates. Additionally, the SOCCS algorithm reduced the rejected VNFs with regards to LLS by 11%, 12% and 12% for 8, 10 and 12 events per unit time, respectively. For the same generation rates, these reductions were higher in comparison with KS, demonstrated by values of up to 16%, 23% and 18%.

#### 5.3.2. Average Acceptance Ratio

**Figure 12:**
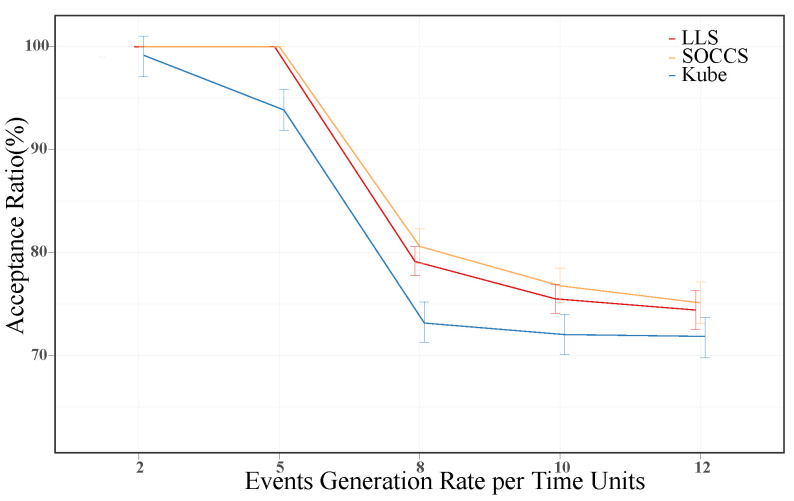
Event acceptance ratio for each scheduling algorithm.

The acceptance ratio represents the proportion between scheduled and requested events. Figure 12 shows its average value for each generation rate. We can see that KS had the worst performance for all the generation rates (e.g., its worst value is above 72%). The other algorithms, LLS and SOCCS, had similar results for low-value generation rates. However, their differences are apparent for arrival rates of 8 or more events per unit of time. Our proposed algorithm increased the acceptance ratio by around 2% with regards to LLS. Thus, SOCCS had the best performance.

#### 5.3.3. Average Number of Successfully Scheduled Events and Deadline Violations

**Figure 13:**
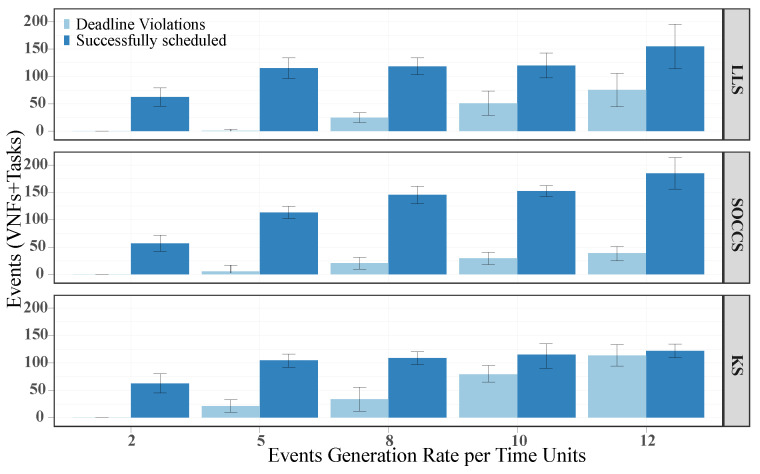
Number of successfully scheduled events and deadline violations for each scheduling algorithm.

Figure 13 illustrates a deeper insight into the number of scheduled events, since it separates the events with deadline violations from the successful ones. In general, our algorithm avoided more deadline violations than the others for all the generation rates. For the highest generation rate (i.e., 12 events per unit time), SOCCS decreased the number of deadline violations by 75% and 83% with regards to LLS and KS, respectively. The exception was for 5 events per unit of time. In this case, LLS had the lowest value.

#### 5.3.4. Average Waiting and Total Time

**Figure 14:**
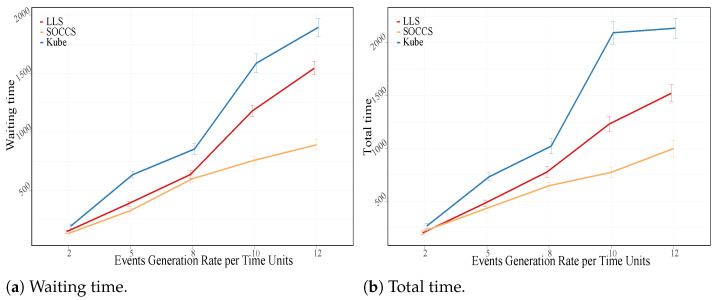
Time metrics for all the scheduling algorithms.

As seen in Section 5.1, the values of the waiting and total times are higher when the scheduler deploys events in the controller node. From Figure 14, we can affirm that SOCCS had the lowest increment for both metrics. Our scheduler reduced the waiting time for the highest generation rate by 42% and 53% with regards to LLS and KS, respectively (see Figure 14a). Additionally, SOCCS also decreased the total time by 34% and 53% in comparison with LLS and KS, respectively (see Figure 14b).

#### 5.3.5. Average Battery Consumption

**Figure 15:**
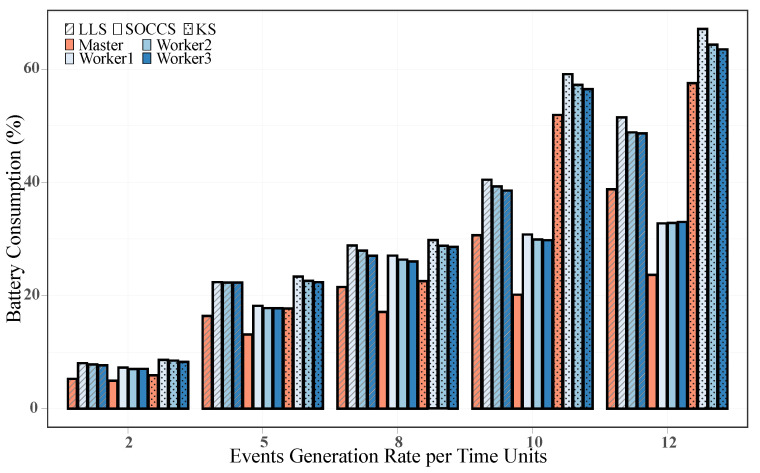
Battery consumption for each node while running different scheduling algorithms.

To obtain the battery consumption, we calculated the difference between the initial and final measured *SOC* in each experiment. Figure 15 depicts the average battery consumption for each node when running different scheduling algorithms to deploy events. In general, the greater the event arrival rate, the higher the battery consumption. By comparing the three algorithms, we can see that the KS had the highest battery consumption for all the generation rates. In contrast, SOCCS guaranteed the lowest battery consumption, as it always selected the node with the highest score to assign the requested event. Namely, it chose the node with the maximum *SOC* and minimum CPU usage. Additionally, the previous subsection revealed that our scheduler performs faster than the baseline algorithms, since the total time of the events in the system was notably reduced. Thus, our scheduler reduced the cluster’s operation time to process the requested events, which indisputably had a lower impact on battery consumption with regards to the other schedulers. By taking a closer look at the highest generation rate, our scheduler is shown to have saved up to 39% and 59% of the battery consumption in the controller node with regards to LLS and KS, respectively. Likewise, it decreased the consumption in Worker1 by 36% and 51% in comparison with the same algorithms. Regarding the workers’ imbalance in terms of battery consumption, we found that our proposed algorithm had the best performance. For a generation rate of 12 events per time unit, SOCCS presented an imbalance of 0.25 between the maximum and minimum average battery consumption. This value was much lower than the ones obtained in the LLS and KS algorithms (2.82 and 3.61, respectively).

## 6. Conclusions

In this paper, we proposed a scheduling algorithm, SOCCS, that assigns events to an SBC cluster by considering predicted battery consumption and used resources. The predictions were obtained through a regression model that establishes the relation between the *SOC* and the CPU usage. The proposed scheduler was implemented and evaluated in a Raspberry Pi cluster running Kubernetes.

Additionally, we analyzed the case of using the unused controller resources to deploy certain services and tasks. The obtained results confirmed that such consideration is a good criterion in a resource-constrained environment. By deploying events in the controller node, we increased the acceptance ratio by around 30% for the highest generation rate with regards to not deploying requests. Similarly, the acceptance ratio increment was related to a significant reduction in the rejected events, which could be up to 66% for a generation rate of 12 events per unit of time. Thus, the proposed scheduler could make better use of the available resources in the cluster.

The evaluation results revealed that the presented solution outperformed the baseline algorithms with regards to analyzed metrics, such as rejected and scheduled events, deadline violations in scheduled events and battery consumption. Specifically, the SOCCS algorithm reduced the rejected VNFs for the highest event generation rate by around 23%, which was evidenced by its high acceptance ratio values. Additionally, our proposed algorithm decreased the number of deadline violations in the scheduled events by 83%. This result was confirmed by the low values in the waiting and total time of the events when the algorithm was used. Finally, our proposed scheduler saved around 59% of battery consumption in the controller node and 51% in the computing nodes for the highest generation rate.

In terms of future work, we intend to provide our proposed scheduler with a mechanism to migrate events and functionalities to other nodes. This strategy would improve the fault tolerance of the cluster, since we could reassign demanding events in critical nodes to available ones in other clusters before the system shuts down due to battery depletion. In this regard, we also need to provide our scheduler with a communication channel to exchange information with other entities with a global network view, such as SDN controllers.

## Figures and Tables

**Table 1:** System model notation.

Notation	Description
*P*	Set of deployable units of computing nodes
*N*	Set of physical nodes where events can be scheduled
$N_{c}$	Set of computing nodes ($N_{c}\subset N$)
*S*	Network service request arriving at a controller node
*F*	Sequence of VNFs compounding a network service request
*T*	Task request arriving at a controller node
*p*	Each virtual node created on the physical nodes to run the events
*n*	Each physical node where virtual nodes are created
$p_{f_{i}}$	Indicates the virtual node where function $f_{i}$ is running
$p_{T}$	Indicates the virtual node where task *T* is running
$f_{i}$	Each network function forming part of a network service
$r_{e}$	Demanded rate of each task and network function
$c_{n}$	Processing capacity of each physical node (n∈N)
$t_{r}$	Running time of an event before considering it completed
$t_{s}$	Starting time of an event when being processed in the selected node
$t_{c}$	Completion time of an event in the selected node
$t_{e}$	Execution time of an event in the assigned node
$t_{a}$	Arrival time of an event request in the controller node
$t_{t}$	Total time of an event in the system
$t_{w}$	Waiting time of an event in the priority queue
*d*	Deadline for processing a given event

**Table 2:** Evaluation parameter ranges based on testbed.

Parameter	Values
Number of VNFs in a service	5–10
Processing capacity per node (MIPS)	500–3000
CPU capacity per node (milliCPU)	4000
Memory capacity per node (Ki)	7,998,464
Required processing rate per event (MIPS)	100–500
Required CPU per event (milliCPU)	150–250
Required memory per event (Ki)	200–500

## Data Availability

The data presented in this study are available on request from the corresponding author. The data are not publicly available due to the public repository is under development.
